# Stamping Monitoring by Using an Adaptive 1D Convolutional Neural Network

**DOI:** 10.3390/s21010262

**Published:** 2021-01-02

**Authors:** Chih-Yung Huang, Zaky Dzulfikri

**Affiliations:** Department of Mechanical Engineering, National Chin-Yi University of Technology, Taichung 41170, Taiwan; zakydzul@gmail.com

**Keywords:** stamping process, vibration, spectrum density, one-dimensional convolutional neural network, classification

## Abstract

Stamping is one of the most widely used processes in the sheet metalworking industry. Because of the increasing demand for a faster process, ensuring that the stamping process is conducted without compromising quality is crucial. The tool used in the stamping process is crucial to the efficiency of the process; therefore, effective monitoring of the tool health condition is essential for detecting stamping defects. In this study, vibration measurement was used to monitor the stamping process and tool health. A system was developed for capturing signals in the stamping process, and each stamping cycle was selected through template matching. A one-dimensional (1D) convolutional neural network (CNN) was developed to classify the tool wear condition. The results revealed that the 1D CNN architecture a yielded a high accuracy (>99%) and fast adaptability among different models.

## 1. Introduction

Stamping remains one of the most widely used processes in sheet metalworking and is the primary process in the manufacture of many products, such as automotive products, electronics, and medical devices. To ensure the consistent quality of the manufactured products, monitoring of the tools used in the stamping process is crucial. Therefore, monitoring data are required to clearly understand factors affecting the conditions of stamping tools [1].

Several studies have focused on monitoring the condition of tools that are used in machining processes such as drilling, turning, and milling; nevertheless, studies that have focused on monitoring the conditions of tools used in stamping processes are few, particularly those involving data acquisition and data recognition or classification. Several studies have proposed the use of force, strain, acoustic emission, vibration, and audio indicators for monitoring the stamping process. However, the analyses executed in these studies did not cover all monitoring processes; instead, such analyses were mostly centered on recognition, classification, or regression processes. Wu et al. [2] proposed using vibration signals to monitor the micropiercing process and logistic regression to predict damage. Sari et al. [3] used acoustic emission to monitor the micropiercing process online. Shanbhag et al. [4] investigated the galling wear of a stamping tool by evaluating acoustic emission frequency characteristics. Ge et al. [5] modeled monitoring signals at different periods in the stamping process by using several autoregressive models with residue as a feature, followed by classification by using the Hidden Markov Model. Tian et al. [6] used machine vision to detect tool surface defects engendered by the stamping and grinding of flat parts.

The strain signal is the most commonly used monitoring signal since it is proportional to the stamping force. However, strain sensors can identify only certain faults and cannot be applied to detect dynamic characteristics of the stamping operation, especially in the high frequency band [7]. On the other hand, AE and vibration force sensors are suitable for detection of the changes in the process and wear. Nevertheless, they are difficult to install and expensive. In terms of vibration sensors, they are easy to acquire and contain ample information about the process dynamics. Although problem of low signal and noise ratio was mentioned before, we applied PCB356A15 accelerometers with sensitivities of 100 mV/g in the present study to solve this issue and clear stamping signals were observed.

Vibration signals provide information crucial in determining dynamic behaviors at high frequencies in the stamping process [7]. Zhang et al. [8] used piezoelectric strain sensors to measure the strain on the press column surface through feature selection to monitor and detect failures in the punching process. Sari et al. [9] demonstrated the relationship between increasing the amplitude within a certain frequency range and the appearance of burrs and onset of damage that leads to punch failure. Continuous use of the tool can cause various problems, such as wear, variation in the quality of the product, and damage to the machine. Cutting tools usually exhibit adhesive and abrasive wear at contact zones [10]. Zhang et al. [11] used the bispectrum to analyze acceleration signals during the online stamping operation, which helped detect tool failure or tool wear.

Vibration signal data captured during the stamping process exhibit different patterns, which can be used to monitor tool health. Some studies have used various pattern matching methods [12,13,14,15] to process vibration signals; the present study implemented these methods for analysis. A template is required as a basis for capturing the vibration signals because different parameters could affect the shape of the signal pattern, resulting in patterns with varying shapes.

Zhao et al. [12] demonstrated the applicability of many deep learning methods to machine monitoring. The success of classical machine learning techniques depends on efficient feature extraction, which requires considerable resources and time. Recently developed techniques in deep learning have enabled automatic feature extraction without the need for an expert, thereby simplifying the final solution while obtaining high accuracy. Recent studies on one-dimensional (1D) signals, especially vibration signals, have used convolutional neural networks (CNNs) [16,17,18,19,20,21] for classification.

On the basis of the findings and limitations indicated by the aforementioned studies, the present study focused on developing a system for capturing vibration signals and a 1D CNN model for classifying such signals to achieve end-to-end monitoring of stamping tool wear. This study’s contributions are detailed as follows.

- The study developed of a system for processing vibration signals by using a template matching algorithm in order to identify tool wear conditions.
- The study applied a deep 1D CNN along with a fully connected neural network (FCNN) for feature extraction to classify tool wear conditions. The optimal configuration was determined through the comparison of 1D CNN and FCNN configurations.
- The study applied real data from a mass production workpiece to evaluate the developed methods and algorithms.
- The proposed method not only enables the reliable and real-time monitoring of stamping tools through processes involving data acquisition and data classification; the method also minimizes computational time throughout these processes. The experimental results demonstrated the applicability of our method to actual machines, with the method exhibiting high signal acquisition rates and excellent classification efficiency.

The remainder of the paper is organized as follows. In Section 2, the proposed methods and data acquisition process are presented. In Section 3, the signal acquisition rate and tool wear classification performance are described. Finally, Section 4 presents the conclusion of this study.

## 2. Materials and Methods

**Figure 1:**
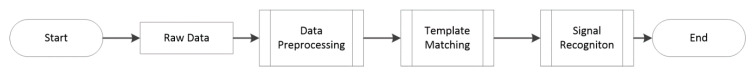
Overall system operation.

The flow of the proposed system’s operation is shown in Figure 1. Raw data obtained from sensors at specific time intervals are first preprocessed and then divided into two signal domains, the time-based domain, and frequency-based domain. After data processing, template matching is used to compare the processed data with the template data. If the required conditions are satisfied, the signal data are selected and then recognized.

### 2.1. Experimental Data

The experimental data used in this study were obtained from an actual working stamping process. Specifically, progressive stamping tools were placed into a reciprocating stamping machine, and an automatic sheet metal feeder fed the metal sheet to be stamped into the stamping tool, which pressed the metal sheet to the intended shape.

**Figure 2:**
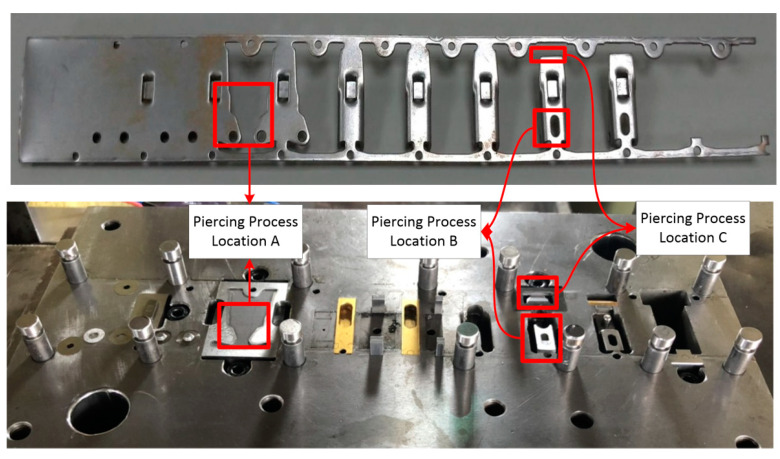
Tool monitoring positions.

**Figure 3:**
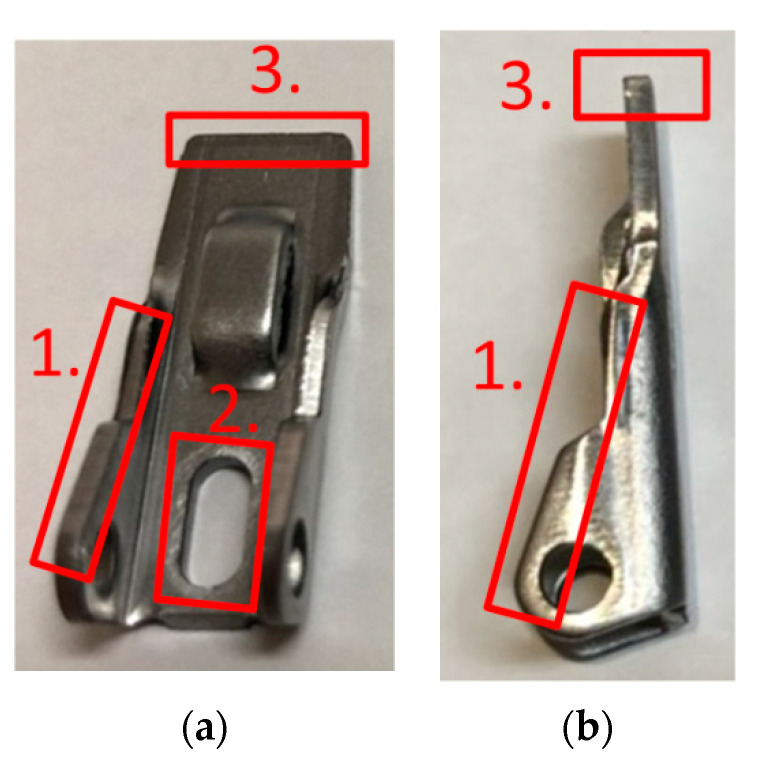
Images of the finished product from the (**a**) top and (**b**) right side; the piercing processes A, B, and C are marked by the numbers 1, 2, and 3, respectively.

**Figure 4:**
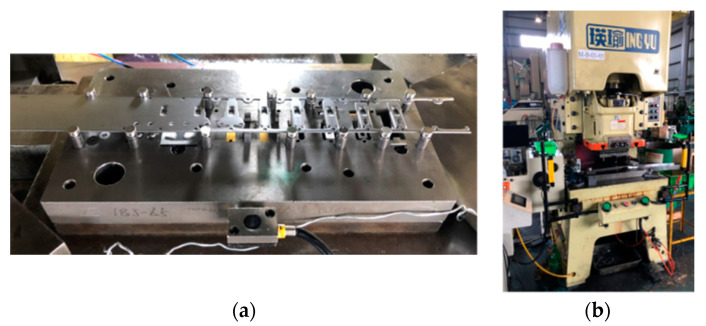
(**a**) tools used and the (**b**) press machine.

Stamping involves various processes, such as blanking, piercing, drawing, forming, and bending, that are executed using distinct tools. In this study, the piercing process, which tends to yield the most tool wear, was monitored at several positions where the tool comes in contact with the material. Figure 2 presents the wear monitoring points inside a progressive tool used for piercing. Figure 3 depicts the finished product, with the numbered boxes indicating the location at which the tool die was applied. The stamping tool, metal sheet workpiece and stamping machine used in this study are illustrated in Figure 4.

After manually inspecting six sets of vibration data from the two sensors, we decided to use the y-axis vibration data from the lower accelerometer because this accelerometer produced distinctive stamping vibration signals and the least data noise. We observed two stages of wear (mild and heavy) at each tool position (Table 1). Detailed conditions are shown in Appendix A. Table 2 presents the classification of the experimental data regarding the various tool conditions, indicating seven class types; only one class represented the healthy condition, three represented the heavy wear condition, and the remaining three represented the mild wear condition. This table also indicates the number of samples and average stamping time for each class.

**Figure 5:**
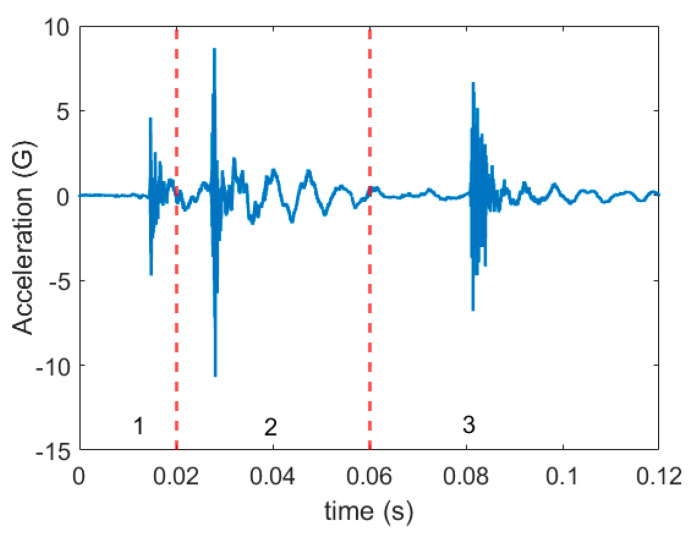
One signal cycle in the stamping process.

In general, in stamping, each vibration signal cycle represents three actions (Figure 5): (1) contact of the stamping tool with the metal sheet, (2) shear force in the metal sheet that is engendered by the impaction of the tool against the metal sheet, and (3) separation of the tool from the metal sheet.

**Figure 6:**
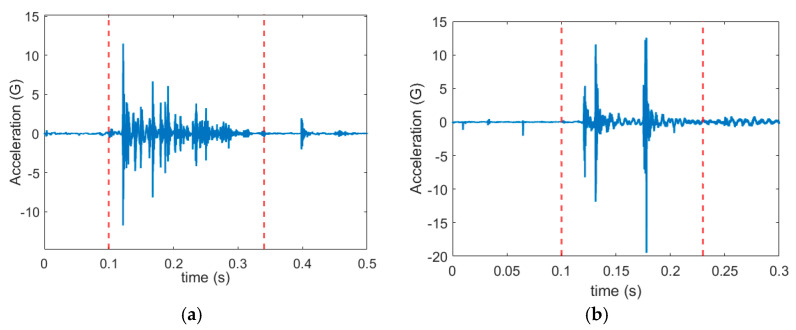
Vibration signals obtained from (**a**) class 4 and (**b**) class 5 in the experimental stamping process.

However, because a real environment was considered in this study, the conditions under which data were extracted could not be adjusted. Therefore, some classes did not include signals that exhibited a typical cycle with the three actions. Instead, shock vibration was produced only when the sheet was cut through shear force. Examples of vibration signals obtained from classes 4 and 5 are illustrated in Figure 6, indicating slight or nonexistent shock vibrations when the tool came into contact with the metal sheet and separated from the metal sheet. Accordingly, we randomly selected a signal with or without action 1 or 3 as the template signal.

### 2.2. Signal Acquisition

**Figure 7:**
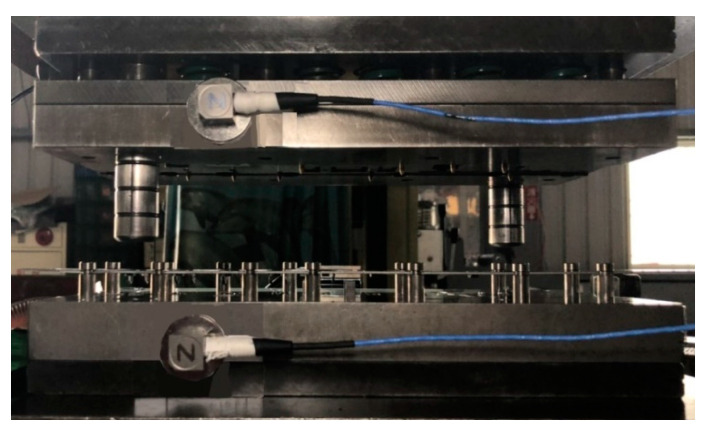
Positions of the accelerometer sensors.

Tool condition data were obtained from an LCP-60H press machine (Ing Yu Machinery, Taichung, Taiwan) with a capacity of 60 tons and an automatic sheet metal feeder. The sheet material was 1.5 mm thick SPCC. Table 3 lists the experimental parameters for the stamping process. Two multiaxis PCB356A15 accelerometers with a sensitivity of 100 mV/g and measurement precision of ±50 g were mounted at the top and bottom sections of the tool (Figure 7) to measure vibrations at a sampling rate of 25,600 Hz; NI 9234 served as the data acquisition module. Throughout the stamping process, signal data were recorded for 10 s whenever a vibration shock was detected; all classes had more than 1 h of raw data on stamping vibrations (comprising more than 92 million data points).

### 2.3. Data Processing

**Figure 8:**
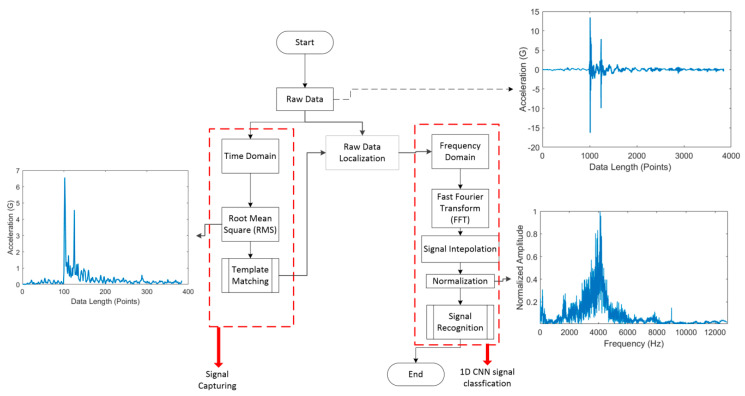
Data processing flowchart.

The obtained vibration signals were then analyzed visually. Figure 8 presents a flowchart of the data preprocessing procedure. This procedure involved two stages: signal acquisition and 1D CNN model execution. For signal acquisition, the raw data were first transformed to a resolution of *n* values by using root mean square (RMS) values to expedite the template matching process. After the acquisition of a signal, the raw data were processed for the 1D CNN model. Such processing is necessary because it prevents extreme input values, which lead to poor results, and because a 1D CNN model cannot process data with inconsistent input lengths. The data set used in this study contained a collection of data objects (samples) whose lengths differed between classes. Because a CNN model requires input data with a fixed length, the data samples were transformed to ensure that they had the same length. Moreover, the power spectral density (PSD) was used as a measure of the signal’s power content versus frequency to account for the possible length variation of the data entered into the system. The frequency range was set to 50% of the sampling rate, which was maintained at 25.6 kHz. We did not split the signal because doing so may obscure meaningful features in the signal itself. In general, the PSD is used to transform time-domain signals of different lengths into frequency-domain signals of the same length. Moreover, it can be used to analyze the frequency of signals x(t) as the ordinary Fourier transform$\;\hat{x}\left(\omega \right)\;$with a finite interval of (0, T). The truncated Fourier transform can be obtained as follows:

(1) $$\hat{x}\left(\omega \right)=\frac{1}{\sqrt{T}}\int_{0}^{T}x\left(t\right)e^{-i\omega t}dt$$

A uniform resolution was not obtained even after the data were transformed to have a fixed frequency range. This problem can be resolved using simple linear interpolation, which is mathematically expressed as follows:

(2) $$\frac{y-y_{0}}{x-x_{0}}=\frac{y_{1}-y_{0}}{x_{1}-x_{0}}$$

The PSD generally represents a very low power amplitude. If such values are entered into the CNN algorithm without normalization (i.e., conversion to a value between 1 and 0), the CNN activation function will not function as intended, thus preventing the model from learning effectively. The normalization equation is expressed in Equation (3):

(3) $$Normalized\;\left(e_{i}\right)=\frac{e_{i}-E_{min}}{E_{max}-E_{min}}$$

### 2.4. Template Matching

To simplify the comparative evaluation of several distance metrics, template matching was used. This technique is designed to be sufficiently simple to avoid confusion and accelerate the computation process.

**Figure 9:**
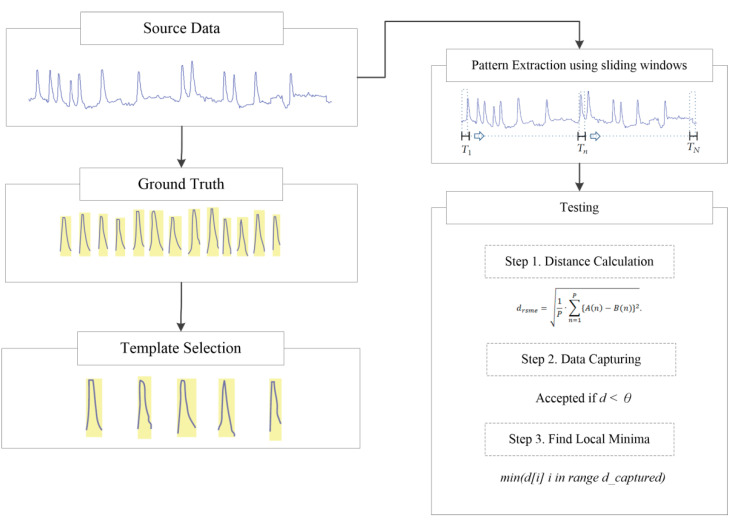
Schematic of the signal matching protocol.

This technique mainly involves calculating the distance between templates and the testing signal and determining the boundary-dependent similarity decisions (Figure 9). In this process, vibration data signals are continuously extracted using sliding windows, which are then converted to a length equal to the length of the template used. Finally, the value of each incoming data point is calculated.

Furthermore, several classes of signals can be extracted. These signals are calculated in terms of distance metrics; specifically, the local minima are calculated to determine the signal that is closest in value to the signal template. The local minima must be smaller than a predetermined threshold value. Signal templates are obtained from manual observations and random selection to avoid possible bias against one method or type of data.

### 2.5. Distance Metrics

This study used several distance metrics, namely, the root mean square error (RMSE), correlation coefficient, kurtosis, skewness, and mean, on the assumption that the shape of the target pattern remained constant. Consider signals *A* and *B* with signal lengths *P_A_* and *P_B_*, respectively. The equations for the distance metrics for these signals are as follows:

(4) $$\begin{array}{c} RMSE:\\ d_{rsme}=\sqrt{\frac{1}{P}\cdot \overset{P}{\underset{n=1}{\sum }}{\left\{A\left(n\right)-B\left(n\right)\right\}}^{2}}. \end{array}$$

(5) $$\begin{array}{c} Correlation\;Coefficient:\\ d_{corr}=\frac{\sum_{n=1}^{P}\left\{A\left(n\right)-\bar{A}\right\}\cdot \left\{B\left(n\right)-\bar{B}\right\}}{\sqrt{\sum_{n=1}^{P}{\left\{A\left(n\right)-\bar{A}\right\}}^{2}}\cdot \sqrt{\sum_{n=1}^{P}{\left\{B\left(n\right)-\bar{B}\right\}}^{2}}}\;. \end{array}$$

(6) $$\begin{array}{c} Kurtosis:\\ d_{kur}=\left|\frac{\sum_{n=1}^{P_{A}}{\left\{A\left(n\right)-\bar{A}\right\}}^{4}}{P_{A}\cdot \sigma_{A}{}^{4}}-\frac{\sum_{n=1}^{P_{B}}{\left\{B\left(n\right)-\bar{B}\right\}}^{4}}{P_{B}\cdot \sigma_{B}{}^{4}}\right|\;. \end{array}$$

(7) $$\begin{array}{c} Skewness:\\ d_{skew}=\frac{\frac{1}{P}\sum_{n=1}^{P}{\left\{\left(A_{n}-B_{n}\right)-\bar{\left(A-B\right)}\right\}}^{3}}{{\left(\sqrt{\frac{1}{P}\sum_{i=1}^{P}{\left\{\left(A_{n}-B_{n}\right)-\bar{\left(A-B\right)}\right\}}^{2}}\right)}^{3}} \end{array}$$

(8) $$\begin{array}{c} Mean:\\ d_{mean}=\frac{\sum_{i=1}^{n}\left(A_{i}-B_{i}\right)}{n} \end{array}$$

*A(n)* and *B(n)* represent the *n*th data points of signals *A* and *B*, respectively, and *P* represent the length of the signals.

### 2.6. Autothreshold

The signal matching protocol (Figure 7) includes a step wherein a threshold is defined for the algorithm to search for the local minima. The algorithm comprises an autothreshold value from the template used, a standard deviation equation for the RSME metric Equation (9), and the variance for the mean distance metric Equation (10). The signals are captured after a comparison between the incoming signals and template signals; if an incoming signal has the same shape and magnitude as the template signal, the value of the distance metric decreases or increases, as discussed in Section 3.1.

(9) $$\begin{array}{c} Standard\;Deviation:\\ \sigma =\sqrt{\frac{\sum {\left(x_{i}-\mu \right)}^{2}}{P}} \end{array}$$

(10) $$\begin{array}{c} Variance:\\ V=\frac{1}{P-1}\overset{P}{\underset{i=1}{\sum }}{\left|x_{i}-\mu \right|}^{2} \end{array}$$

$x_{i}$ = value from the template signal; μ = mean value of the template signal; P = template signal length.

### 2.7. Convolutional Neural Network

**Figure 10:**
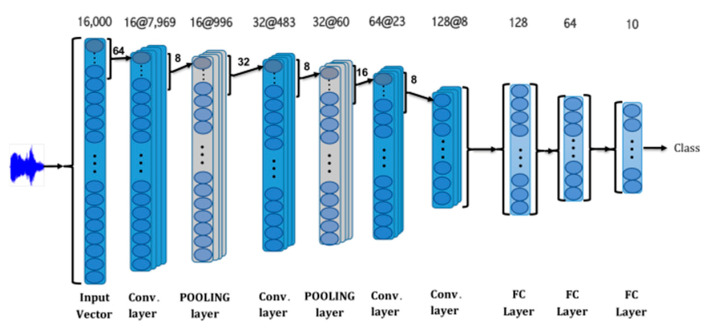
1D convolutional neural network (CNN) with a fully connected layer [12].

Deep CNNs are designed to be operate on 2D datasets of images and videos; however, 1D CNNs have clear advantages over 2D CNNs for capturing signals. Like 2D CNNs, 1D CNNs also serve as trainable filters that can learn to filter important features from 1D data. The output from 1D CNNs can then be used in an FCNN (Figure 10) to perform conventional classification operations. 1D CNNs differ from 2D CNNs in that they use a 1D array for feature maps and kernels; hence, the kernel size and parameters for subsampling are in the form of scalars. The FCNN uses the conventional backpropagation (BP) method.

1D forward propagation from convolution layer *l* − 1 to the input of a neuron in layer *l* is expressed as shown in Equation (11).

(11) $$x_{k}^{l}=b_{k}^{l}+\overset{N_{l-1}}{\underset{i=1}{\sum }}conv1D\left(w_{k}^{l-1},s_{k}^{l-1}\right)$$

where $x_{k}^{l}$ represents the input, $b_{k}^{l}$ is the bias of the *k*th neuron at layer *l*, and $s_{k}^{l-1}$ is the output of the *i*th neuron at layer *l* − 1. Moreover, $w_{k}^{l-1}$ represents the kernel (“mask” or “weight”) from the *i*th neuron at layer *l* − 1 to the *k*th neuron at layer *l*. Therefore, if $y_{k}^{l}$ is defined as the output, it can be expressed by a function of $x_{k}^{l}$, as shown in Equation (12):

(12) $$\begin{array}{c} y_{k}^{l}=f\left(x_{k}^{l}\right)\\ s_{k}^{l}=y_{k}^{l}↓ss \end{array}$$

where $s_{k}^{l}$ denotes an output of the *k*th neuron in the *l* layer and ↓ ss denotes a downscaling factor for the output with a scale factor of ss. In an FCNN, BP is performed starting from the multilayer perceptron if it has *N_L_* classes. The mean square error (MSE) is expressed as follows:

(13) $$E_{p}=MSE\left(t^{p},\left[y_{1}^{L},\ldots ,y_{N_{L}}^{L}\right]\right)=\overset{N_{L}}{\underset{i=0}{\sum }}{\left(y_{i}^{L}-t_{i}^{p}\right)}^{2}$$

where L=1 represents the input layer, l=L represents the identity between an input and output, p represents the input vector, $t_{i}^{p}$ represents the corresponding target, and $\left[y_{1}^{L},\ldots ,y_{N_{L}}^{L}\right]$ represent the output vectors.

In this study, batch normalization (BN) was used after the application of the convolution filters. Suppose that the data contain m training examples; the mean can be calculated using Equation (14):

(14) $$\mu_{ℬ}\leftarrow \frac{1}{m}\overset{m}{\underset{i=1}{\sum }}x_{i}$$

Then, the variance of these training examples or the mini batch can be calculated as follows:

(15) $$\sigma_{ℬ}^{2}\leftarrow \frac{1}{m}\overset{m}{\underset{i=1}{\sum }}{\left(x_{i}-\mu_{ℬ}\right)}^{2}$$

After the variance is obtained, the formula for BN can be applied, as shown in Equation (16), where γ and β are trainable parameters.

(16) $$y_{i}\leftarrow \gamma \frac{x_{i}-\mu_{ℬ}}{\sqrt{\sigma_{ℬ}^{2}+\epsilon }}+\beta =BN_{\gamma ,\beta }\left(x_{i}\right)$$

Moreover, LeakyReLU nonlinearity can be used, as expressed in Equation (17):

(17) $$f\left(x\right)\;=\;\{\begin{array}{c} x,\;\;if\;x>0\\ ax,\;otherwise \end{array}$$

**Figure 11:**
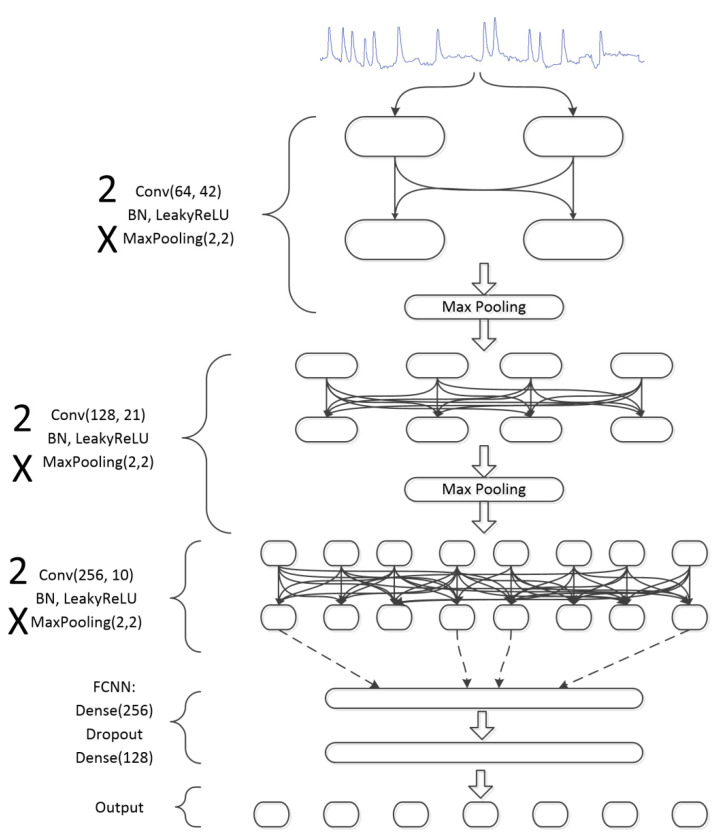
Diagram of a convolutional network with attached fully connected neural network (FCNN).

The reference model of 1D CNN used in this study is shown in Figure 11. It combines convolutional filters, batch normalization, following by performing LeakyReLU and MaxPooling to attain downsampling.

## 3. Results

The main results of this study pertained to two aspects, namely, signal acquisition and signal recognition. Results pertaining to signal acquisition revealed the system’s signal acquisition accuracy, and those pertaining to signal recognition revealed the 1D CNN model’s performance in classifying the stamping signals. Template matching were conducted using the 64-bit version of LabVIEW 2019, whereas 1D CNN modeling was conducted using Tensorflow in Python. These systems were run on a Windows computer with an AMD Ryzen 7 3750H processor, 24 GB of RAM, and NVIDIA GeForce RTX 2060.

### 3.1. Distance Metrics Performance

Five algorithms were compared with respect to two key indicators. The first indicator was the distinctive distance, which was evaluated in the absence of a signal, in the presence of a noise signal, and in the presence of a stamping signal.

**Figure 12:**
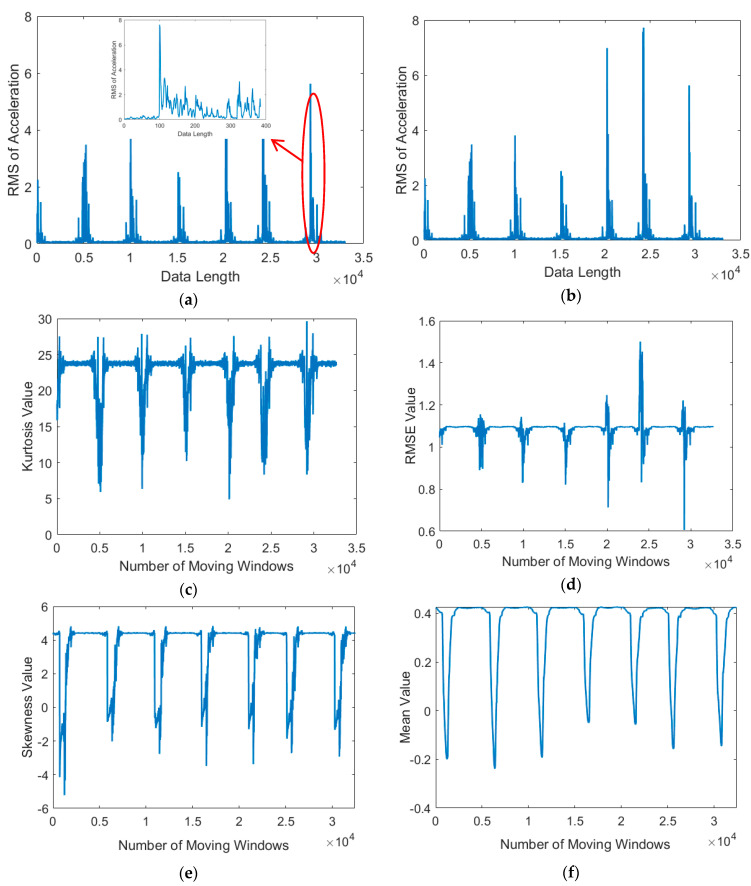
(**a**) Root mean square (RMS) of signals from the vibration in the stamping process. Sliding windows were applied from the template signal across the RMS signal, resulting in (**b**) the correlation coefficient, (**c**) kurtosis, (**d**) RMSE, (**e**) skewness, and (**f**) mean metrics.

Figure 12 presents the performance of each distance metric. All three algorithms could detect vibration signals to some degree, and the kurtosis, RMSE, skewness, and mean values indicated that the algorithms could distinguish between time points with a noise signal, without a signal, and with a stamping signal. However, the correlation coefficient tends to fluctuate with a small change in input value. This is because the calculation of the correlation coefficient ignores the magnitude and only focuses on the “shape” of each vector. Therefore, the correlation coefficient is unsuitable as a distance metric for signal matching after acquiring a vibratory signal because determining the optimal threshold value would be difficult. The second indicator was the computation time.

**Figure 13:**
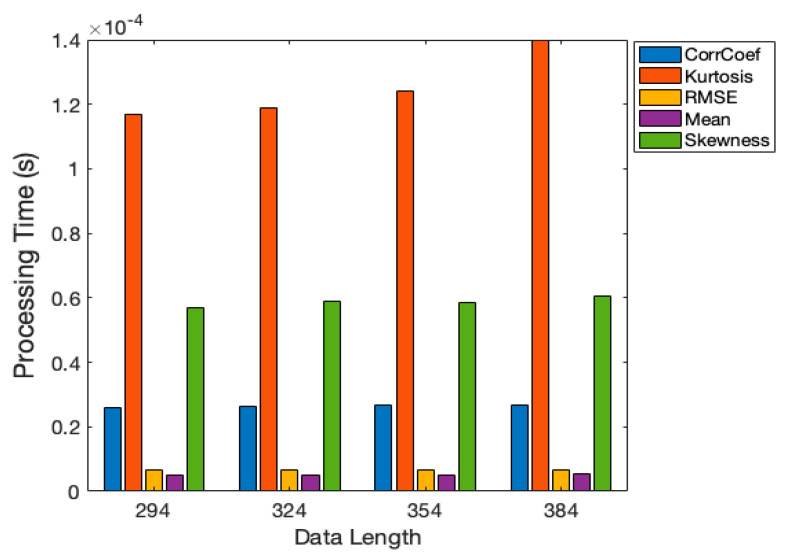
Distance metrics: computation time.

The vibration signal obtained in the stamping process could be as short as 0.1 s. Therefore, to capture such short signals, the computation time should be measured for each distance metric in order to determine the metric that yields the shortest computation time. Figure 13 presents the computation time of each distance metric. As expected, the RMSE and mean had the shortest computation time because only simple mathematical operations are used. Although the correlation coefficient, kurtosis, and skewness had short computation times, they required the data length to be scaled, which lengthened the computation time. Accordingly, the RMSE and mean was indicated the be most suitable (i.e., best performing) distance metric.

### 3.2. Autothresholding Performance

**Figure 14:**
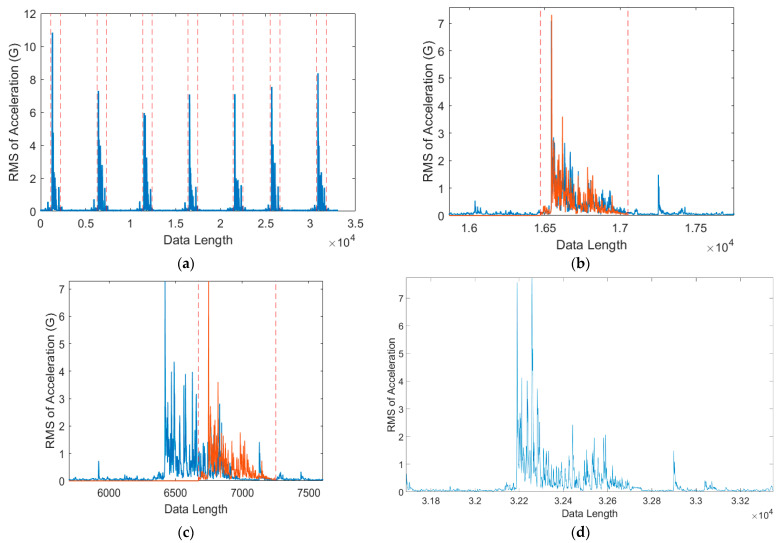
(**a**) Results of signal acquisition using autothresholding. Red dotted lines indicate the best matched signal. (**b**) True positive signal, (**c**) false positive signal, and (**d**) and false negative signal.

In this study, autothresholding performance was tested in terms of the RMSE and mean. These tests involved assessments of sensitivity or recall, miss rate, and precision or positive predicted value. In this experiment, template signals of random size were manually selected from random sections in each class; the selection process was repeated four times in each class. All the signals were obtained when the tool cut the metal sheet. Three measures were used: true positive (TP), false positive (FP), and false negative (FN). Figure 14b shows a TP classification; that is, the algorithm could fully capture a signal. Figure 14c presents an FP classification; that is, the algorithm failed to capture the whole signal, with one or more parts missing. Figure 14d shows an FN classification; that is, the algorithm could not capture the signal of interest. The TF signal is not presented in Figure 14 because it is only applicable in the condition when no stamping signal is present.

**Figure 15:**
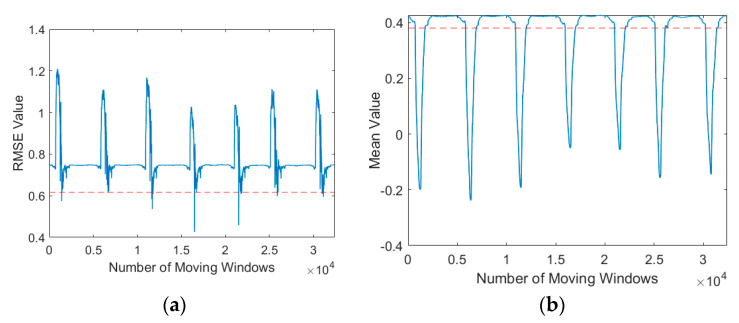
Autothresholding performance as assessed in terms of (**a**) standard deviation and (**b**) variance of the template signal (red dotted line).

**Figure 16:**
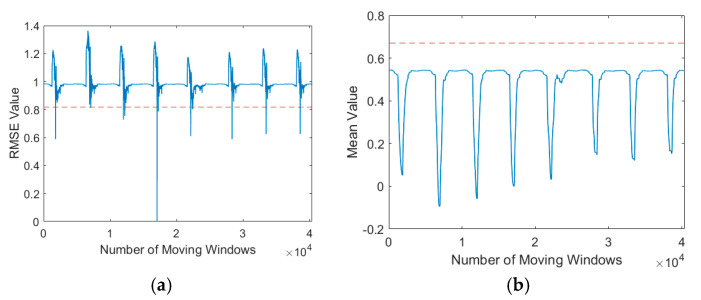
Autothresholding using (**a**) standard deviation and (**b**) variance in same template signal.

Figure 15 shows the autothresholding performance as assessed in terms of the standard deviation of the RMSE and the variance of the mean. The thresholds are indicated by the red dotted line, and the RMSE and mean values were obtained using sliding windows. This figure indicates that autothresholding could be used to determine whether local minima should be calculated. This reduces computation time while ensuring that the stamping signal is captured. Although the standard deviation of the RMSE and variance of the mean can be used as the autothreshold, we found some inconsistency when using the variance as the autothreshold (Figure 16) for some cases where the template signal was long; if the variance autothreshold is below its mean value, local minima cannot be detected.

Thus, we only used the RMSE as the distance metric and the standard deviation as the autothreshold.

**Figure 17:**
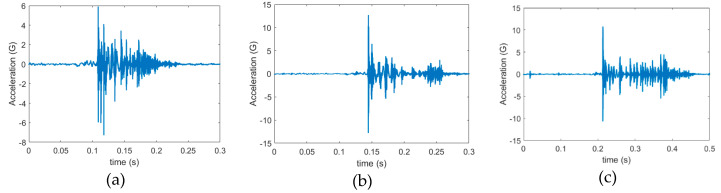

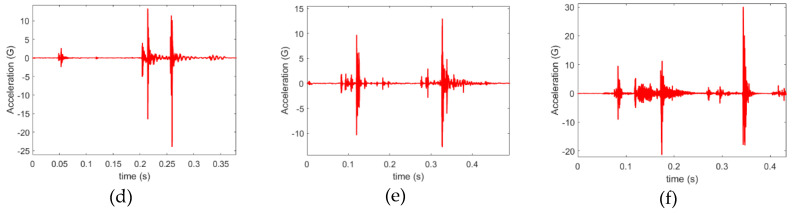
Vibratory signal from (**a**)–(**f**) represent class 2 to 7, respectively.

We observed that classes with the same degree of wear also had the same type of signal in terms of shape and amplitude (Figure 17); heavy but not mild wear was associated with continuous vibration to some degree. We conducted an autothresholding experiment by using class 1 (the healthy condition), by randomly selecting one of classes 2 to 4 (heavy wear conditions), and by randomly selecting one of classes 5 to 7 (mild wear conditions). Table 4 shows the results for data from the same class, and cross-class. Detailed results are shown in Appendix B. The results revealed a high sensitivity (0.950) and precision (0.876). Cross-class examination is required because, in real-time monitoring, the incoming signals can be from any class. Table 4b presents the results of cross-class examination, meaning that a template from another class is used to capture signals from other classes. The sensitivity and precision exceeded 0.8 in some cases but were very low for other cases. An investigation was conducted for cases with poor precision, and these cases were found to involve very narrow template signals, which resulted in very narrow windows for the acquisition of the correct signal, resulting in poor sensitivity and precision.

### 3.3. Tool Condition Classification Using a 1D CNN and Model Optimization

**Figure 18:**
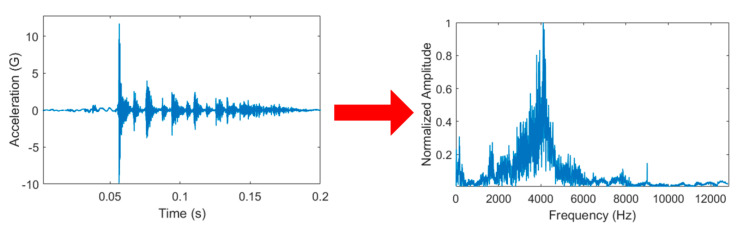
Normalized transformation of Fast Fourier Transform from time-based to frequency-based.

The proposed CNN was tested using data obtained according to the description in Section 2.1. These input data were then preprocessed to have the same data length by using the method described in Section 2.3 (Figure 18).

**Figure 19:**
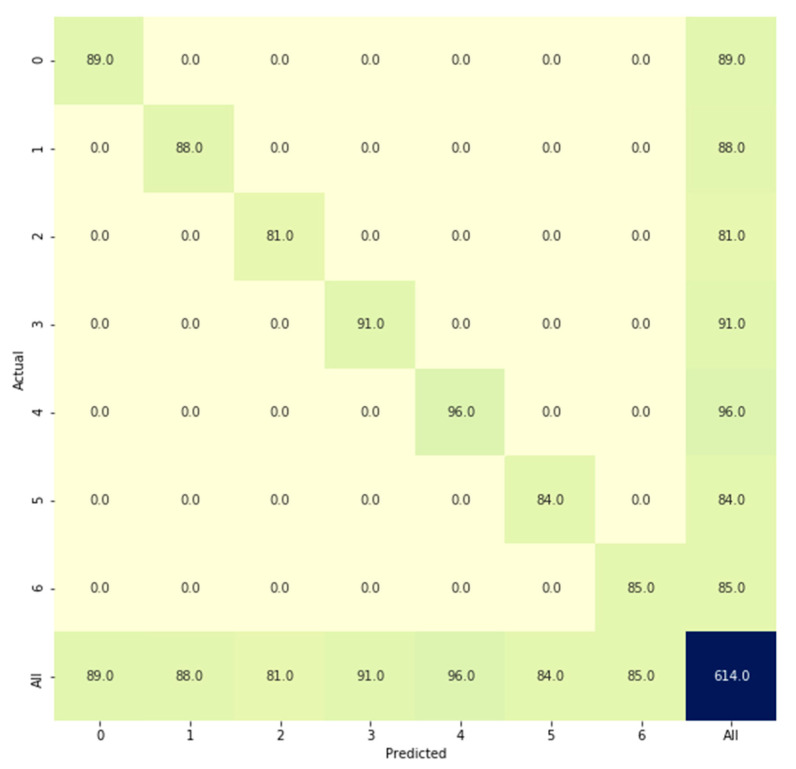
Confusion matrix of the classification results.

**Figure 20:**
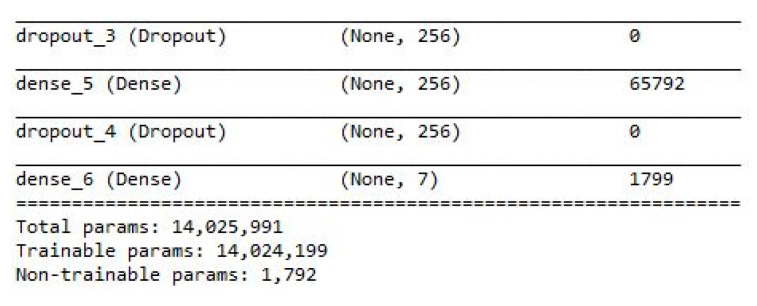
Total numbers of parameters from the reference model.

Figure 19 presents the classification results produced by the model (referenced in Section 2.7) when testing data were used. For every class, the predicted values matched the actual values. We could analyze misclassifications using a confusion matrix; this matrix was also used to evaluate classification accuracy. An accuracy rate of 100% was obtained, but many previous studies have reported that adaptive 1D CNNs can perform better than other classification algorithms, such as support vector machine (SVM), k-nearest neighbors, SVM ensemble, and artificial neural network architectures. In this study, hyperparameter optimization was used because the model must be as simple as possible to reduce the computation time while maintaining a high accuracy for classifying each class correctly. The total numbers of parameters obtained by the reference model described in Section 2.7 are shown in Figure 20. The parameters comprised weights and biases that were either trainable or not trainable in the 1D CNN model.

The number of nodes in the FCNN and CNN filters was reduced to decrease the number of parameters, but other hyperparameters, such as the alpha value for LeakyReLU and the dropout, were not altered because they did not significantly reduce the size of the model. Of the data available, 30% and 70% were validation and training data, respectively. The results obtained after the training process indicated significant differences between models with respect to the total number of parameters (Table 5). Although the number of parameters decreased sequentially from the reference model to the smallest model, the accuracy did not decrease significantly. Furthermore, the second smallest model with 200,000 parameters exhibited a high level of accuracy; although, it had only 1/137 of the parameters in the reference model; however, when that model had only 100,000 parameters, the accuracy decreased, and we thus did not decrease the number of parameters further.

### 3.4. Data Reduction for Model Training

**Figure 21:**
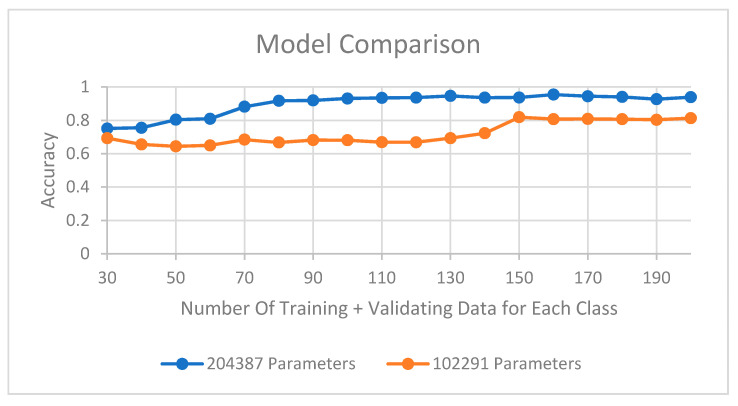
Comparison of accuracies for different amounts of training data.

The case in this study only used one type of progressive stamping tool; however, in actual case scenarios, the proposed model architecture could be used for other types of stamping tools. Figure 21 illustrates a graph that can serve as a reference for determining the quantity of data required to retrain the model. The graph depicts the behavior of the two smallest models when they were trained using different quantities of data. The data shown in the figure include data that were used for training and validation; the remaining data were used for testing. Because the accuracy of each model was tested using the test data, these data had not been used during model training. As stated in Section 3.3, the model with 200,000 parameters yielded the lowest accuracy, which can be confirmed by the results shown in Figure 21.

The majority of one cycle monitoring were composed by signal acquisition, data preprocessing, and prediction using 1D CNN. In the present study, the actual computation times of one cycle monitoring period of the 1960 samples were 0.14–0.18 s, and it depended on the data length of template signal. For example, in this study case, the stamping speed was 40 rpm which equals to 1.5 s of one cycle stamping process time. The computation times of one cycle monitoring period was 0.18 s; therefore, there was enough time to real-time monitoring and classify the condition of tool wear during every cycle stamping process. The proposed method could be applied for stamping speed up to 100 rpm and satisfy the industrial specifications of most metal stamping.

## 4. Conclusions

In this study, a method of capturing vibratory signals during the stamping process was proposed and verified using real-case data. Several distance metrics were compared, and the results revealed that the RMSE was the most suitable distance metric due to its effectiveness and speed. An autothresholding method was also employed to help determine the local minimum threshold from different template signals. The proposed method was validated based on sensitivity and precision. A sensitivity of >0.9 (indicating favorable performance) was obtained from experimental results for the same-class and cross-class configurations, and a precision of >0.85 was obtained for both configurations. The results of this study can serve as a reference for the extraction of signals with periodic characteristics and shape similarity.

The use of an adaptive 1D CNN for classifying stamping-induced vibration signals was extensively studied. Localized raw data of vibration signals were normalized using the PSD and then entered into the 1D CNN Model. The 1D CNN presents the advantages of adaptability and rapid feature learning. Moreover, the effectiveness of the 1D CNN was assessed using a real-case scenario. The size of the model in this study was reduced greatly owing to the rapid adaptability of the 1D CNN, and an accuracy of up to 99.8% was achieved. The amounts of training and validation data were also investigated. An accuracy of >90% could be achieved with only 90 data points from each class. These findings show that the 1D CNN can be used in real time for classifying vibration signals during stamping. Although the effectiveness of the proposed 1D CNN was verified, problems remain to be addressed for its application in mass production. The proposed 1D CNN is not flexible in terms of the number of classes. Future studies should aim to overcome this problem by using Siamese neural networks.

This study’s findings can serve as a reference for manufacturers in developing strategies for reducing the cost of tool maintenance and improving product quality.

## Figures and Tables

**Table 1:** Tool wear figure and name tagging.

Identification Number	Process Location	Mild Wear	Heavy Wear
1.	Piercing Position A	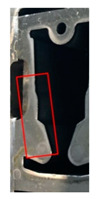	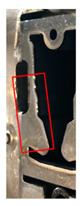
2.	Piercing Position B	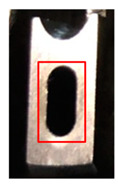	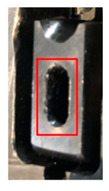
3.	Piercing Position C	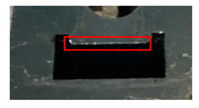	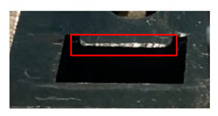

**Table 2:** Data obtained from the experiment.

Class Name	Class Type	Number of Samples	Average Stamping Time (s)
Healthy Condition	Class 1	280	0.26
Heavy Wear Position A	Class 2	280	0.183
Heavy Wear Position B	Class 3	280	0.27
Heavy Wear Position C	Class 4	280	0.393
Mild Wear Position A	Class 5	280	0.113
Mild Wear Position B	Class 6	280	0.13
Mild Wear Position C	Class 7	280	0.12

**Table 3:** Experimental parameters for the stamping process.

Lubrication	Anti-Corrosive Oil
Sheet material	SPCC
Sheet thickness (mm)	1.5
Stamping speed (rpm)	40
Die material	SKD 11
Average Blanking force	29.5 kN
Number of parts formed	1960

**Table 4:** Average sensitivity, precision, and miss rates for data acquisition (a) within the same class and (b) cross various classes.

(a)				(b)			
Class	Sensitivity Rate	Precision	Miss Rate	Class	Sensitivity Rate	Precision	Miss Rate
1	1	0.892	0	1→4	1	0.892	0
3	1	0.821	0	3→1	0.922	0.815	0.077
7	0.851	0.917	0.148	7→2	0.797	0.866	0.202
Average	0.95	0.876	0.049	Average	0.9067	0.8583	0.093

**Table 5:** Model size and accuracy.

Total Parameters	Accuracy (%)
14,025,991	100
13,040,311	99.8
13,026,135	99.8
3,260,943	99.8
3,257,007	99.8
1,631,599	99.8
817,231	99.8
204,495	99.7
204,387	99.8
102,291	98.0

**Figure A1:**
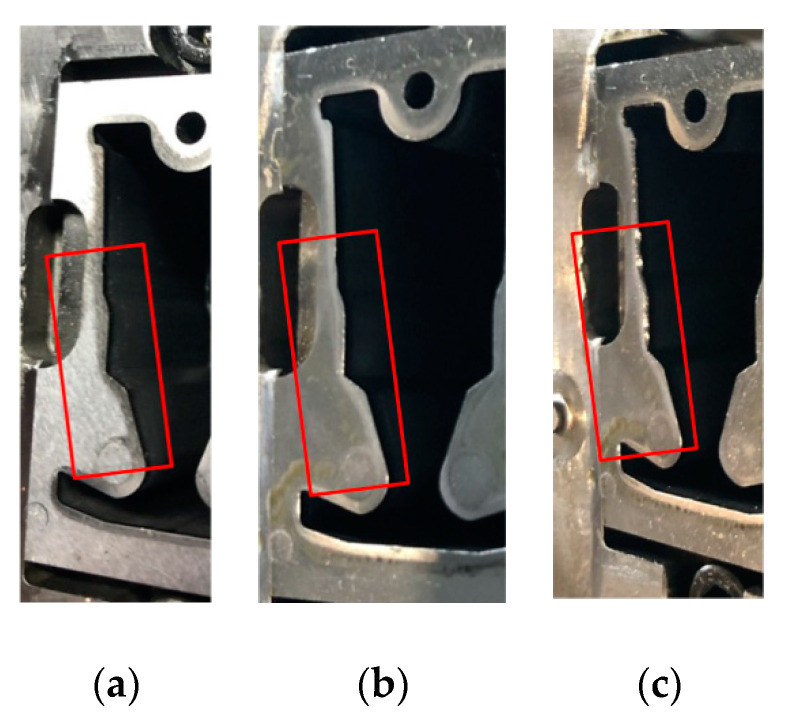
(**a**) Healthy condition, (**b**) mild wear, and (**c**) heavy wear at position A.

**Figure A2:**
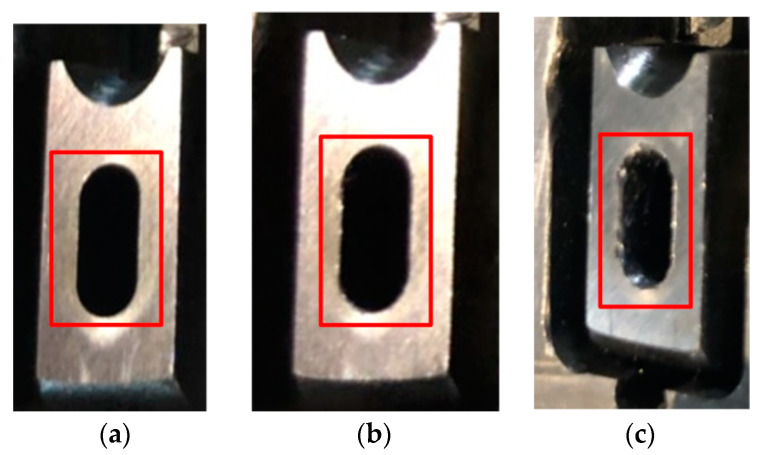
(**a**) Healthy condition, (**b**) mild wear, and (**c**) heavy wear at position B.

**Figure A3:**
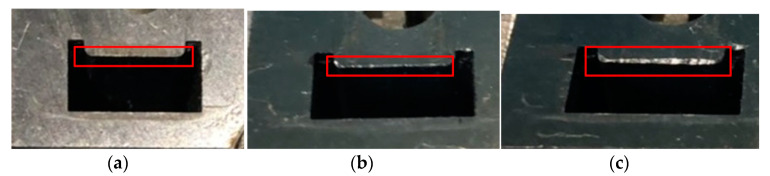
(**a**) Healthy condition, (**b**) mild wear, and (**c**) heavy wear at position C.

## Data Availability

The data presented in this study are available on request from the corresponding author. The data are not publicly available due to further study will be carried out using the same data.

## Appendix B

**Table A1:** (a) True positive signal, (b) false negative signal, (c) false positive signal.

**(a)**				
**Sensitivity Rate**				
**Class**	**Trial**			
	**1**	**2**	**3**	**4**
**1**	1	1	1	1
**3**	1	1	1	1
**7**	0.714	1	0.833	0.857
**(b)**				
**Precision**				
**Class**	**Trial**			
	**1**	**2**	**3**	**4**
**1**	1	0.857	0.857	0.857
**3**	0.857	0.857	0.857	0.71
**7**	0.833	1	0.833	1
**(c)**				
**Miss Rate**				
**Class**	**Trial**			
	**1**	**2**	**3**	**4**
**1**	0	0	0	0
**3**	0	0	0	0
**7**	0.285	0	0.166	0.142

**Table A2:** (d) sensitivity, (e) precision, (f) and miss rate for cross-class capture. “1→4” means that signals were obtained from class 1 and templates were from class 4.

(**d**)				
**Sensitivity Rate**				
**Class**	**Trial**			
	**1**	**2**	**3**	**4**
**1→4**	1	1	1	1
**3→1**	0.857	1	1	0.833
**7→2**	0.833	0.857	0.833	0.667
(**e**)				
**Precision**				
**Class**	**Trial**			
	**1**	**2**	**3**	**4**
**1→4**	0.857	1	0.857	0.857
**3→1**	1	0.428	1	0.833
**7→2**	0.833	1	0.833	0.8
(**f**)				
**Miss Rate**				
**Class**	**Trial**			
	**1**	**2**	**3**	**4**
**1→4**	0	0	0	0
**3→1**	0.142	0	0	0.167
**7→2**	0.167	0.142	0.167	0.333
